# Management of shoulder dystocia: a re-audit

**DOI:** 10.1186/1753-6561-6-S4-P11

**Published:** 2012-07-09

**Authors:** N Lynch, C Emmerson

**Affiliations:** 1 Medical School, Newcastle University, UK

## Introduction

Shoulder dystocia is an obstetric emergency with raised neonatal morbidity including brachial plexus injury. With much litigation the Clinical Negligence Scheme for Trusts (CNST) advise minimum standards for documentation.

## Objectives

To audit compliance against documentation standards, analyse the manoeuvres used to resolve shoulder dystocia and quantify brachial plexus injuries.

## Methods

Follow-up of a 2009 audit. Cases between April 2010 and January 2011 were identified from the birth register and incident reports. Documentation was analysed against the minimum standards. Manoeuvres used to resolve each case were examined. The risk manager confirmed which cases resulted in brachial plexus injury.

## Results

There were 23 cases of shoulder dystocia; one case had to be discounted. Documentation had declined since 2009 when all areas were 100% documented. Only 65% of cases recorded the manoeuvres used, their timing and stage of delivery. The CNST additionally requires the sequence of manoeuvres and who conducted them which was not routinely recorded. McRobert's position resolved 50% of cases, the remainder escalated to suprapubic pressure (14%), entry manoeuvres (18%) and posterior arm removal (18%). Only 41% recorded the staff attending and the time they arrived. The rate of brachial plexus injury rose from 6.7% to 15.8%.

## Conclusions

Documentation is poor in several areas with two major CNST requirements not being met. The pro forma must be updated to capture the necessary details. Skill drills should be re-commenced in the department to minimise the risk of brachial plexus injury.

